# Acute Cerebral Infarct and Saddle Pulmonary Embolism in a Post-COVID-19 Patient Treated With Thrombolytics

**DOI:** 10.7759/cureus.33877

**Published:** 2023-01-17

**Authors:** Christopher Sampson, Obiaara Ukah

**Affiliations:** 1 Emergency Medicine, University of Missouri School of Medicine, Columbia, USA

**Keywords:** embolism, stroke, case report, thrombolytics, covid-19, pe, cva

## Abstract

The coronavirus disease 2019 (COVID-19) has been associated with a prothrombotic state during active infection with the severe acute respiratory syndrome coronavirus (SARS-CoV-2). However, reports of delayed multisystemic COVID-19-associated thromboembolic sequelae are limited in the current literature. In addition, the initiation of prophylactic antithrombotic therapy in patients for the prevention of such events during and after infection remains controversial due to conflicting reports. Here, we present evidence to support that patients with comorbid conditions are at higher risk for multisystemic COVID-19-associated thromboembolic events and propose that beginning prophylactic therapy in this population could lead to more favorable outcomes. We describe a 67-year-old male who presented with left-sided weakness and worsening shortness of breath and was diagnosed with COVID-19 approximately one month prior. Initial computed tomography (CT) of the brain showed an age-indeterminate cerebellar infarct. On CT angiography (CTA) of the neck, a saddle embolus was incidentally found and was confirmed on an immediate follow-up chest CT. After consultation with neurology, a decision was made to administer thrombolytics at the standard recommended stroke dosing. The patient was admitted to the ICU and received mechanical thrombectomy two days later. At the time of hospital discharge, the patient still had left-sided weakness on examination and required no additional oxygen support. This report reviews the prevalence of delayed sequelae of COVID-19 and the benefit of prophylactic antithrombotic therapy during active and post-SARS-CoV-2 infection. It is important for emergency medicine physicians to recognize that patients who have recovered from COVID-19 are at high risk for delayed thromboembolic disease, even in the absence of personal or family history of coagulopathy. This underscores the necessity of obtaining imaging studies in high-risk patients who present with acute symptoms that cannot be explained by other probable causes. In addition, patients should be encouraged to follow up with their primary care providers to discuss prophylactic anticoagulation therapy as it could be beneficial during and post-COVID-19.

## Introduction

Infection of severe acute respiratory syndrome coronavirus 2 (SARS-CoV-2), the etiological cause of coronavirus disease 2019 (COVID-19), typically presents with fever and respiratory symptoms that include dry cough, dyspnea, and hypoxia in moderate cases and more severe pulmonary disease in severe cases. In addition to respiratory compromise, SARS-CoV-2 infection has been shown to affect the neurological, gastrointestinal, renal, cardiovascular, and hematological systems [1]. Thromboembolic complications can occur during acute infection or may be delayed. In addition, the benefit of prophylactic anticoagulation therapy during and after infection remains controversial in even the most vulnerable of patients with comorbidities such as diabetes mellitus (DM) and cardiovascular disease [2]. Here, we present the case of a patient with comorbid illness with COVID-19 sequelae of a subacute, likely embolic, infarction of the right cerebral hemisphere and an incidental finding of a large saddle embolus in the right and left main pulmonary arteries who presented with left-sided weakness and numbness in addition to worsening shortness of breath. Our case is unique in that we describe a comorbid patient with no previous history of coagulopathy who was found to have venous thrombotic disease that progressed to multisystemic arterial thromboembolic disease one month after an active SARS-CoV-2 infection. This case demonstrates the importance of having a high index of suspicion for delayed vascular thrombosis in acutely symptomatic patients, especially those at high risk with comorbidities, after an active SARS-CoV-2 infection. In addition, it supports the importance of considering anticoagulation therapy during and post-recovery in high-risk patients.

## Case presentation

A 67-year-old male with a past medical history significant for hypertension, hyperlipidemia, type 2 diabetes mellitus, hypothyroidism, and recovery from severe COVID-19 infection presented to the emergency department (ED) approximately three hours after family members observed that the patient had left-sided facial droop with left-sided upper and lower extremity weakness and worsening shortness of breath. The patient tested positive for COVID-19 three weeks prior to this presentation using a home test. A week prior to this ED visit, the patient was clinically diagnosed with COVID-19 pneumonia, requiring 5 L of oxygen via nasal cannula at baseline at home due to exacerbated respiratory symptoms. The course of treatment for his pneumonia was unknown because the patient could not provide the information and was from outside of our health system. The patient also reported recently being restricted to reclined seating due to oxygen desaturations that occurred when rising from sitting positions or ambulating at home. There were no other clear precipitating or alleviating factors for reported symptoms. The patient denied all other reviews of systems, including fever, chills, cold sweats, fatigue, and malaise.

The patient’s vital signs on presentation were unremarkable apart from requiring a FiO2 of 100% and an oxygen flow rate of 35 L/minute on a heated high-flow nasal cannula to achieve a pulse oximetry saturation of 99%. Without oxygen supplementation, pulse oximetry would fall below 90%. Other benign findings included a temperature of 36.4°C, heart rate near the upper limit of normal at 92 beats/minute, respiratory rate of 19 breaths/minute, and blood pressure of 138/86 mmHg. The patient’s physical examination was notable for moderate acute distress secondary to respiratory failure. He had a National Institutes of Health (NIH) Stroke Scale score of 10 with left-sided facial droop, 1/5 strength in the left upper and lower extremities, left-sided numbness, and left-sided extinction.

The patient’s laboratory results revealed a complete blood count with leukocytosis of 18.49 × 10^9^/L (reference range: 3.50-10.50 × 10^9^/L) with mild anemia noted by hemoglobin of 12.3 g/dL (reference range: 13.5-17.5 g/dL). The comprehensive metabolic panel was otherwise unremarkable apart from mild hyponatremia at 133 mmol/L (reference range: 136-145 mmol/L), hyperglycemia at 377 mg/dL (reference range: 70-139 mg/dL), and hypoalbuminemia at 2.8 g/dL (reference range: 3.5-5.2 g/dL). Coagulation studies were unremarkable with partial thromboplastin time (PTT) of 31.9 seconds (reference range: 25.8-35.6 seconds), international normalized ratio (INR) of 1.2 (reference range: 0.9-1.2), and slightly elevated prothrombin time (PT) of 16.2 seconds (reference range: 12.6-15.1 seconds). Brain natriuretic peptide resulted in 3,585 pg/mL (reference range: 0-125 pg/mL).

**Figure 1:**
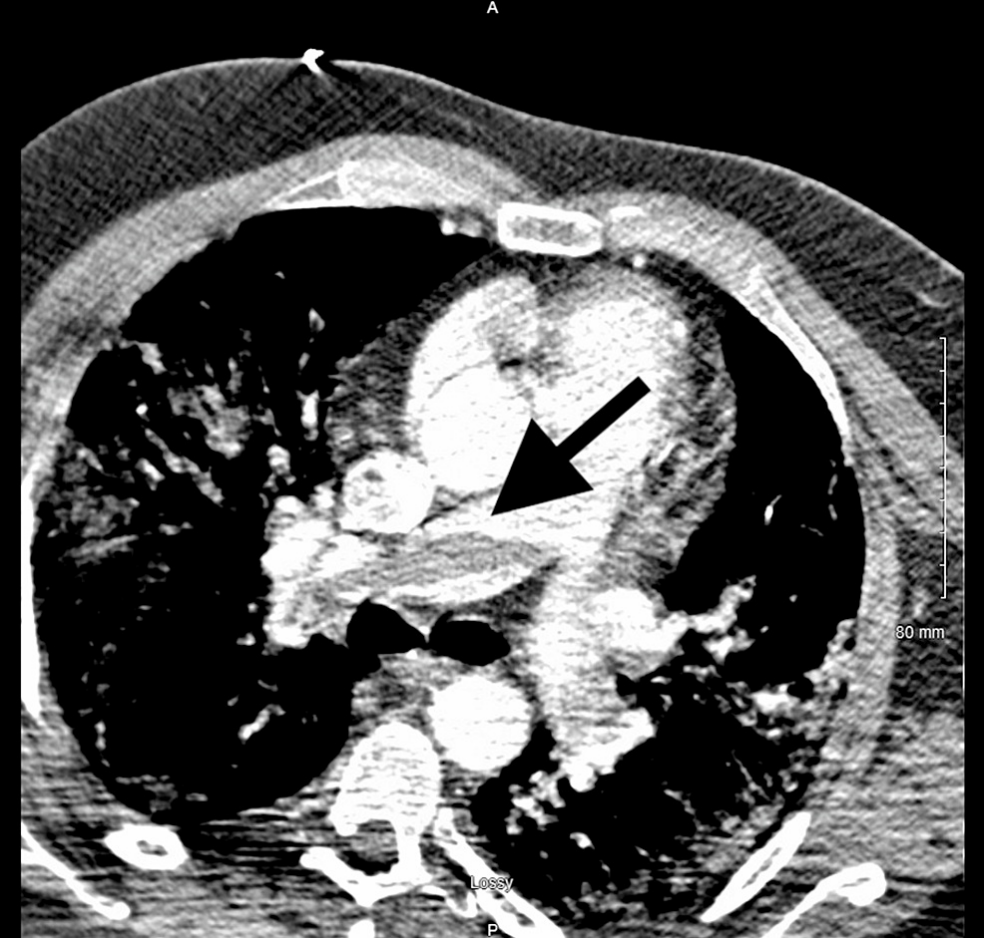
CT showing saddle embolus in the pulmonary artery (arrow) CT: computed tomography

Due to the patient’s presenting symptoms of left-sided numbness and weakness, axial computed tomography (CT) of the head, CT perfusion of the head, and CT angiography (CTA) of the head and neck studies were ordered. CT of the head revealed an age-indeterminate infarction, likely embolic, in the right cerebellar hemisphere, but no acute intracranial bleed. CT perfusion of the head showed possible penumbra in the right occipital lobe region without any underlying cerebral blood flow or cerebral volume abnormality, indicating ischemic tissue that would benefit from reperfusion therapy [3]. CTA of the head and neck revealed no intracranial or neck arterial stenosis or occlusion. Surprisingly, CTA of the neck incidentally revealed a large saddle embolus in the right and left main pulmonary arteries (Figure 1).

A subsequent CT of the chest with pulmonary embolism (PE) protocol confirmed the presence of the saddle embolus and revealed diffuse bilateral consolidative and ground glass airspace opacities that were concerning for COVID-19 pneumonia. After a discussion between emergency medicine and neurology, the patient was administered a tissue plasminogen activator (tPA) because he presented to the ED within the recommended stroke time frame, continued to have persistent stroke symptoms, and had increasing oxygen requirements. It was felt that thrombolytics would treat both emergent conditions. A decision was made to use the stroke dosing of alteplase, which based on the patient’s weight was administered as 9 mg intravenous push, followed by 81 mg as an intravenous infusion. A pulmonary thrombectomy was temporarily postponed for the reassessment of clot burden after tPA administration. The patient was then admitted to the neurological intensive care unit (NICU). Inpatient ultrasonography revealed occlusive deep vein thromboses (DVTs) in the right popliteal vein and left posterior tibial and peroneal veins, and a nonocclusive DVT in the right superficial femoral vein.

A mechanical thrombectomy for the saddle pulmonary embolism was successfully performed on hospital day 2. Postoperatively, the patient’s supplemental oxygen demands improved significantly; he required only 6 L/minute via nasal cannula and was weaned to 2 L/minute over one week. He was also placed on a heparin drip and then transitioned to apixaban before discharge to an inpatient rehabilitation facility two weeks later. At the time of discharge, the patient still have 3/5 power in the left upper and lower extremities but required no additional oxygen for respiratory support.

## Discussion

There is evidence to suggest that multisystemic thrombosis may occur as a complication of COVID-19 weeks or even months after initial infection and recovery. Previously reported cases presented patients with simultaneous brain infarcts, pulmonary embolisms, and other vascular anomalies unique to each case, while any other probable causes for thrombotic disease other than diabetes were ruled out [4,5]. In our case, the patient developed pneumonia, likely secondary to COVID-19, and had comorbidities of hypertension, hyperlipidemia, and type 2 diabetes mellitus, which placed him at high risk for thrombosis. Although the patient had no history of coagulopathy, he developed bilateral lower extremity DVTs that presumably led to a cerebral infarct and massive saddle pulmonary embolism approximately one month after contracting COVID-19. Whether he previously tested positive via polymerase chain reaction (PCR) or serology was unknown as outside records were unable to be obtained, but he tested positive again via PCR prior to admission to the NICU.

It is estimated that 10%-30% of individuals who test positive via PCR continue to have positive PCR results one month later, especially if they had severe COVID-19, have comorbidities, and/or are immunosuppressed or elderly [6]. It is likely that our patient’s positive PCR result is from his initial infection weeks prior and his presentation of multisystemic thrombosis is sequelae of COVID-19. This is important to consider because prior to admission, our patient developed respiratory symptoms that could have been due to continued SARS-CoV-2 infection rather than a bacterial or non-COVID-19 viral pneumonia. It was approximately one week later that he experienced an exacerbation of respiratory symptoms secondary to his massive saddle embolism.

The mechanism by which COVID-19 promotes hypercoagulability has not been fully elucidated, but it has been shown to involve catastrophic feedback and feedforward loops that induce a hyperinflammatory state [7]. This is mediated by inflammatory cytokine-producing cells, such as neutrophils and T-lymphocytes that are activated by cytokines released from SARS-CoV-2-infected cells [7,8]. The cytokine storm produced in the hyperinflammatory state also activates the Janus kinase (JAK)-signal transducer and activator of transcription (STAT) signaling cascade in endothelial and inflammatory cells [7]. This leads to an upregulation of tissue and thrombotic factors that contribute to the activation of an extrinsic coagulation cascade and, thus, vascular thrombosis [1,7]. Hyperinflammation has also been shown to lead to excessive complement activation that is mediated by the interaction of SARS-CoV-2 with the host cell receptor, angiotensin-converting enzyme 2, and subsequent upregulation of angiotensin II [9]. Complement activation can also lead to the overexpression of tissue factors and significant deposition of complement components in the microvasculature, which also contributes to a prothrombotic state [9,10]. Ongoing research efforts for understanding the mechanisms of COVID-19-associated thrombosis are important for pinpointing key laboratory markers of hypercoagulability and novel potential targets of anticoagulative therapy in patients.

While it is well documented that the thromboembolic complications of SARS-CoV-2 infection are sequelae of patients with severe COVID-19, the administration of prophylactic anticoagulation therapy has been debated because of conflicting reports regarding whether this is of any benefit to patients. A retrospective study of 449 patients with severe COVID-19 demonstrated that the administration of prophylactic heparin reduced mortality compared to untreated controls (40% versus 64%, respectively) [11]. Alternatively, a retrospective study of 2,773 hospitalized patients reported no significant difference in mortality among those treated with anticoagulation during their hospitalization compared to those who did not receive treatment (22.5% versus 22.8%, respectively) [12]. Our patient was not on an anticoagulative for any preexisting conditions, and to our knowledge, he was not prescribed prophylactic therapy by his primary care physician after testing positive for COVID-19.

Our patient had preexisting risk factors for thromboembolism without personal or family history of coagulopathy. The induction of his coagulopathic state was attributed to concomitant infection or sequelae of COVID-19; however, we cannot strongly establish this as the causal event. A recent retrospective study showed that the odds of having a pulmonary embolism in a patient with COVID-19 versus a non-infected patient did not significantly differ, which raises some doubt about the association of COVID-19 with a prothrombotic state [13]. This conflicts with recent literature putting the risk of DVT and PE in the 30 days post-COVID-19 infection at a fivefold and 33-fold increase, respectively [14]. We must take into account that our patient reported being bound to a recliner for approximately one week. This alone placed him at high risk for DVT and could have been the sole cause of his multiple thrombotic events with no correlation to his prior SARS-CoV-2 infection.

## Conclusions

We report a case of a comorbid patient with multisystemic thrombosis, consisting of a right cerebral infarct and massive pulmonary saddle embolism, as sequelae of COVID-19 approximately one month post initial infection. Prophylactic anticoagulation treatment may have prevented this patient’s thrombotic events, but the benefit of therapy, even in those who are at high risk of thrombosis due to comorbidities, remains controversial in current literature. As efforts continue toward deciphering the mechanisms by which a prothrombotic state is induced by SARS-CoV-2 infection, therapies targeted to more specific processes have been implemented. These therapies may be more efficacious and increase the number of individuals, especially comorbid patients at higher risk, who would benefit from a prophylactic anticoagulant. Nevertheless, emergency medicine physicians should continue to have a high suspicion of life-threatening thromboembolic events when patients present with acute, unrelenting symptoms that cannot be explained by other probable causes after recovery from COVID-19.
